# Supplementary material for: Identification and Validation of Ikaros (IKZF1) as a Cancer Driver Gene for Marek’s Disease Virus-Induced Lymphomas
Source: Microorganisms. 2022 Feb 9;10(2):401. doi: 10.3390/microorganisms10020401 (PMC8877892; doi:10.3390/microorganisms10020401)
Supplement: Supplementary file 1 [file microorganisms-10-00401-s001.zip › microorganisms-1437795-supplementary.pdf]

microorganisms

MDPI

*Article*

# Identification and Validation of Ikaros (*IKZF1*) As a Cancer Driver Gene for Marek's Disease Virus-Induced Lymphomas

**Alec Steep [1,†], Evin Hildebrandt [2,‡], Hongen Xu [3,§], Cari Hearn [2], Dmitrij Frishman [3], Masahiro Niikura [4], John R. Dunn [5], Taejoong Kim [5], Steven J. Conrad [5], William M. Muir [6,7] and Hans H. Cheng [2,*]**

[1] Genetics Program, Michigan State University, East Lansing, MI 48824, USA; alec.steep@gmail.com (A.S.)
[2] Avian Disease and Oncology Laboratory, US National Poultry Research Center, Agricultural Research Service, USDA, East Lansing, MI 48823, USA; evin.hildebrandt@gmail.com (E.H.); cari.hearn@usda.gov (C.H.)
[3] Genome-Oriented Bioinformatics, Technical University of Munich, Munich, D-80333, Germany; hongen_xu@hotmail.com (H.X.); d.frishman@wzw.tum.de (D.F.)
[4] Faculty of Health Sciences, Simon Fraser University, Burnaby, BC V5A 1S6, Canada; masahiro_niikura@sfu.ca
[5] Endemic Poultry Viral Diseases Research, US National Poultry Research Center, Agricultural Research Service, USDA, Athens, GA 30605, USA; john.dunn@usda.gov (J.R.D.); taejoong.kim@usda.gov (T.K.); steven.conrad@usda.gov (S.J.C.)
[6] Department of Animal Sciences, Purdue University, West Lafayette, IN 47907, USA; wmmuir@icloud.com
[7] Genesys Bioinformatics Service, Punta Gorda, FL 33950, USA
* Correspondence: hans.cheng@usda.gov
† Current address: Department of Human Genetics, University of Michigan Medical School, Ann Arbor, MI 48109, USA
‡ Current address: Zoetis Animal Health, Kalamazoo, MI 49007, USA
§ Current address: Precision Medicine Center, Academy of Medical Sciences, Zhengzhou University, Zhengzhou, Henan, China

**Supplementary Materials:**

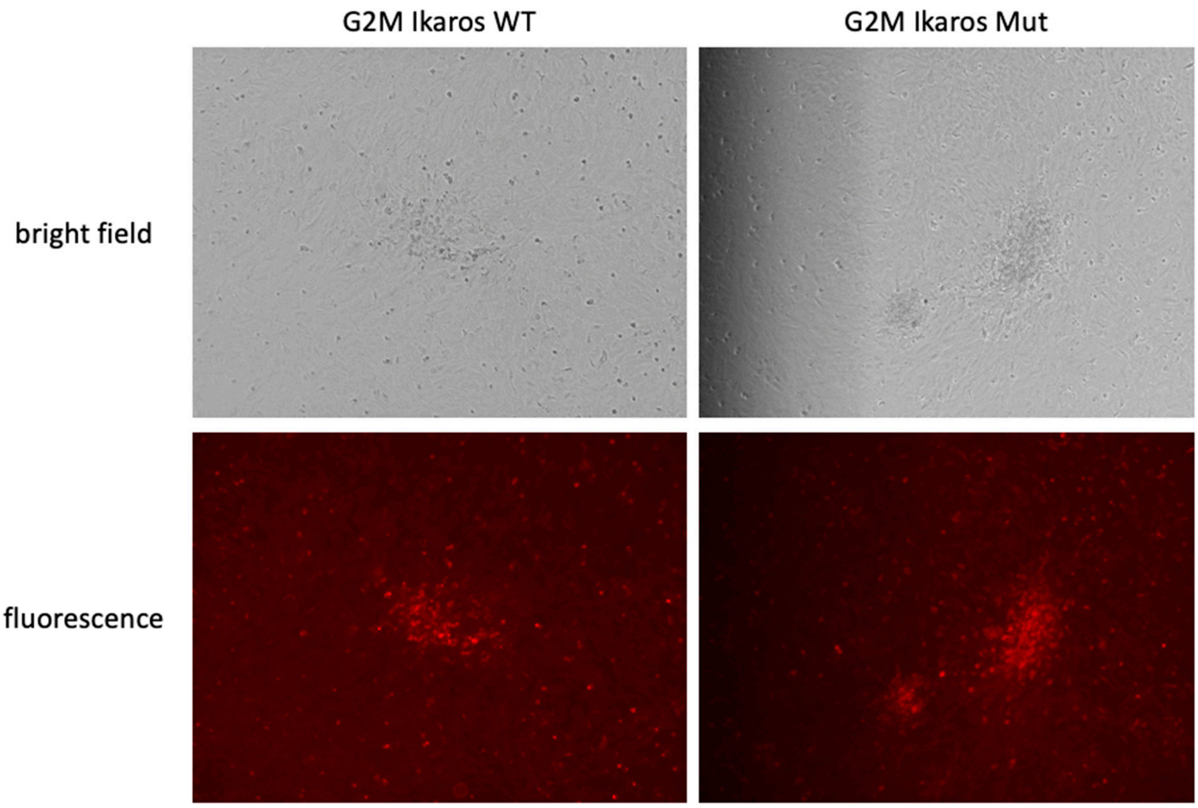

**Figure S1.** Bright field and immunofluorescent staining of G2M Ikaros WT and G2M Ikaros Mut to detect viral plaques and correspondence with expression of Ikaros-FLAG fusion using anti-FLAG tag antibody.

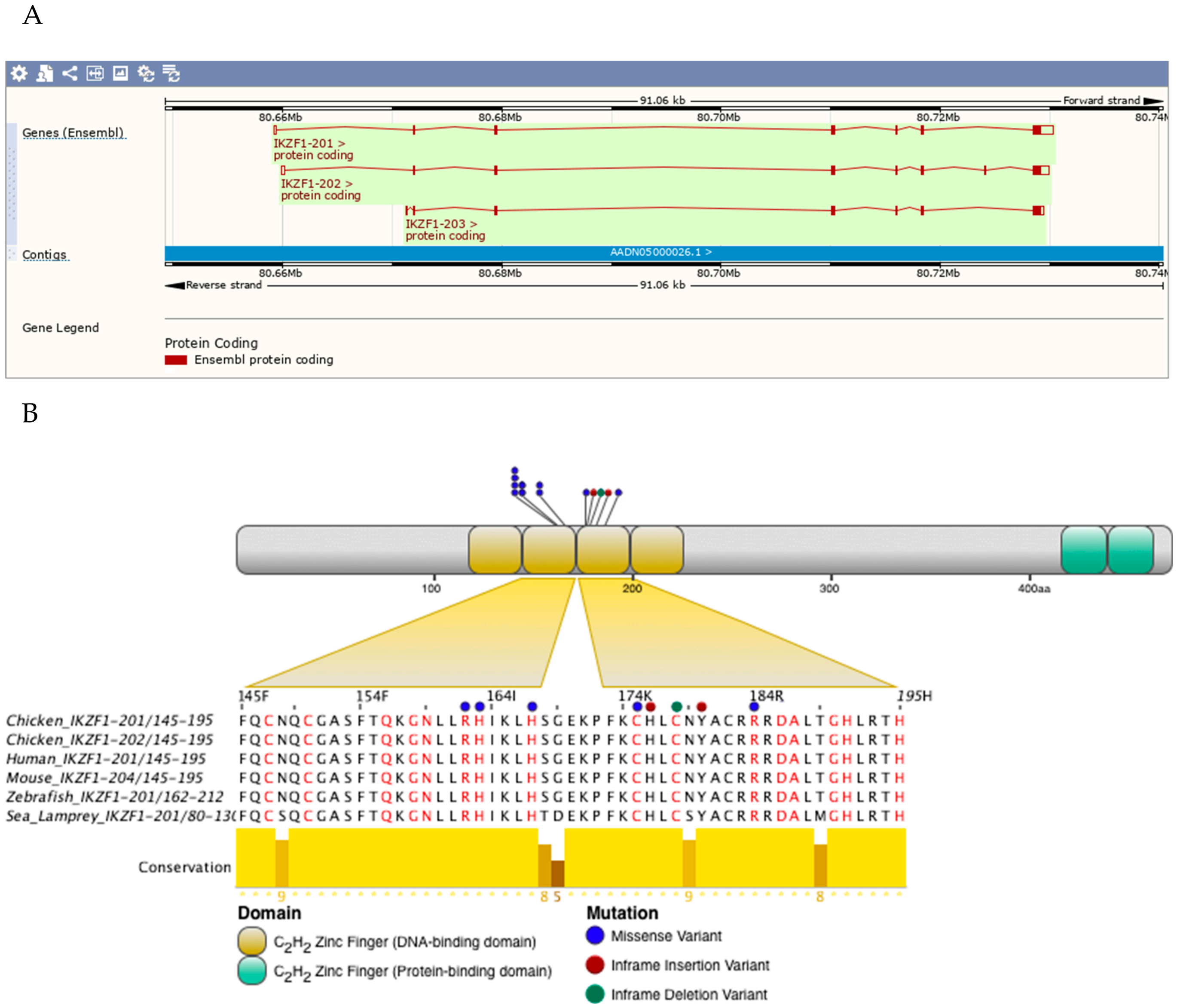


**Figure S2.** (**A**). Ensembl chicken IKZF1 gene information (ENSGALG00000013086) showing the orientation of the exons and the three known expressed isoforms. (**B**) The Ikaros protein with somatic non-synonymous mutations mapped on to the conserved amino acid sequences of DNA-binding zinc fingers 2 and 3. Amino acids essential for DNA-binding [66] are colored red, and conservation scores are represented as yellow bars plots under amino acid sequences. Somatic mutations are represented as colored dots (red, blue, and green).

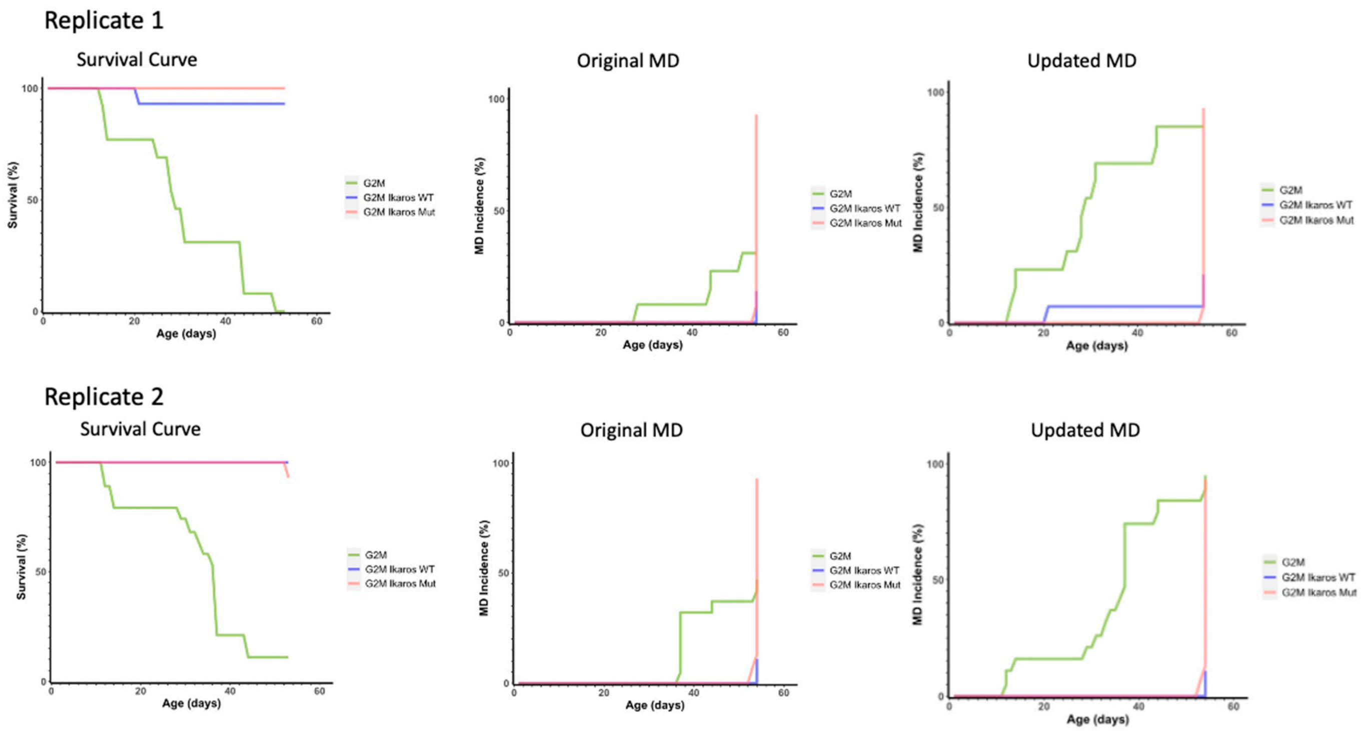


**Figure S3.** Comparison of survival and MD incidence over time for both replicates of bird experiment 2. The columns of the left give survival curves. The middle columns plot MD incidence over time using the traditional definition of MD that includes only birds with tumors or enlarged nerves at necropsy. The columns on the right show MD incidence over time that was expanded to include birds at least 7 days of age that died or developed clinical disease, and had bursa or thymic atrophy (BTA) with a score of 3 or higher (0 to 4 scale) at necropsy.

**Table S1.** MD incidence in comparing the viruses generated from the parental B40 BAC clone with viruses generated from G2M containing only a single repeat long (RL).

| Replicate | Treatment | Total Birds | MD[1] | |
|---|---|---|---|---|
| | | | Count | Percent |
| 1 | none | 5 | 0 | 0 |
| | deltaRL G2M | 16 | 16 | 100 |
| | rMd5 B40 | 15 | 15 | 100 |
| 2 | none | 5 | 0 | 0 |
| | deltaRL G2M | 16 | 16 | 100 |
| | rMd5 B40 | 17 | 16 | 94 |

[1] MD is considered positive when a bird has enlarged nerves or evidence of a tumor.

**Table S2.** Distribution of tumors and nerve enlargements across organs for each recombinant MDV.

**Replicate 1**

| Treatment | No. of birds | No. of birds with tumors | No. of tumors per bird (if present) | Organ with tumor | | | | | | |
|---|---|---|---|---|---|---|---|---|---|---|
| | | | | proventriculus | gonad | spleen | kidney | heart | liver | pancreas |
| none | 6 | 0 | 0 | 0 | 0 | 0 | 0 | 0 | 0 | 0 |
| G2M | 13 | 2 | 3, 3 | 0 | 2 | 0 | 1 | 2 | 1 | 0 |
| G2M Ikaros WT | 14 | 2 | 1, 1 | 0 | 0 | 0 | 0 | 1 | 1 | 0 |
| G2M Ikaros Mut | 14 | 12 | 1, 2, 2, 2, 2, 2, 3, 3, 3, 3, 3, 5 | 2 | 1 | 8 | 0 | 11 | 8 | 1 |

| Treatment | No. of birds | No. of birds with nerve enlargement | Organ with nerve enlargement | | |
|---|---|---|---|---|---|
| | | | vagus | brachial | sciatic |
| none | 6 | 0 | 0 | 0 | 0 |
| G2M | 13 | 4 | 4 | 3 | 1 |
| G2M Ikaros WT | 14 | 1 | 0 | 0 | 1 |
| G2M Ikaros Mut | 14 | 6 | 6 | 2 | 1 |

**Replicate 2**

| Treatment | No. of birds | No. of birds with tumors | No. of tumors per bird (if present) | Organ with tumor | | | | | | |
|---|---|---|---|---|---|---|---|---|---|---|
| | | | | proventriculus | gonad | spleen | kidney | heart | liver | pancreas |
| none | 10 | 0 | 0 | 0 | 0 | 0 | 0 | 0 | 0 | 0 |
| G2M | 19 | 7 | 1, 1, 1, 1, 2, 2, 2 | 0 | 2 | 2 | 0 | 4 | 2 | 0 |
| G2M Ikaros WT | 19 | 2 | 1, 1 | 0 | 0 | 0 | 0 | 2 | 0 | 0 |
| G2M Ikaros Mut | 15 | 14 | 1, 1, 1, 2, 2, 2, 2, 2, 2, 3, 3, 3, 3, 3 | 2 | 1 | 7 | 1 | 12 | 7 | 0 |

| Treatment | No. of birds | No. of birds with nerve enlargement | Organ with nerve enlargement | | |
|---|---|---|---|---|---|
| | | | vagus | brachial | sciatic |
| none | 10 | 0 | 0 | 0 | 0 |
| G2M | 19 | 9 | 9 | 6 | 3 |
| G2M Ikaros WT | 19 | 0 | 0 | 0 | 0 |
| G2M Ikaros Mut | 15 | 3 | 3 | 3 | 2 |
